# Comparing the Quality of Life of Patients Requesting Dental Implants Before and After Implant

**DOI:** 10.2174/1874210601711010485

**Published:** 2017-08-31

**Authors:** Naser Sargolzaie, Amir Moeintaghavi, Hamid Shojaie

**Affiliations:** 1 Dental Research Center, Mashhad University of Medical Sciences, Mashhad, Iran; 2 Oral and Maxillofacial diseases Research Center, Mashhad University of Medical Sciences, Mashhad, Iran

**Keywords:** Quality of life, Dental implants, Edentulous patient, WHO definition for health, Quality of life (QOL), Oral impact on daily practice (OIDP)

## Abstract

**Background and Objectives:**

Tooth loss is a serious life event that impairs two important functions, namely, eating and speaking, and has significant side effects on different aspects of quality of life. These effects are internalized by the individual. The present study aimed to compare the quality of life (QOL) of patients requesting dental implants before and after implant.

**Materials and Methods:**

This analytical cross-sectional study was conducted on patients referred to the Mashhad faculty of Dentistry and private clinics with dental implants in 2015. Patient Quality Of Life (QOL) was assessed using the Oral Impact on Daily Practice (OIDP) questionnaire. Data were analyzed using SPSS software.

**Results:**

In this study, the most common problems reported by patients were eating (78%), smiling, laughing, and embarrassment (53%) before surgery. The quality of life associated with eating; speaking clearly; clean teeth or dentures; light physical activities, such as working at home, going out to work or meeting others; smiling; laughing; showing teeth without discomfort and embarrassment; emotional conditions, such as becoming upset quicker than usual, enjoying communication with others (*i.e.*, friends, relatives and neighbors); and job-related activities significantly increased after surgery, but QOL associated with the amount of sleep and resting did not improve. No significant association was noted between quality of life after implantation and place of residence, education and gender.

**Conclusion:**

In this study, implants had a favorable impact on a patient’s quality of life.

## 1 INTRODUCTION

Quality of life includes conditions that enable good living, such that a person is able to perform everyday activities in a good physical, mental and social state and the patient is satisfied with therapeutic efficacy, disease control, or rehabilitation. The World Health Organization (WHO) provides the following definition for health: complete physical, mental and social well-being, which not only means the absence of disease and disability but also includes body, mind and society axes. Thus, any disability and damage to any of these three axes disrupts the individual’s balance and leads to a lack of health. Following this definition, the international community's attention to the concept of quality of life (QOL) has increased [1]. Orofacial changes, such as diseases and pain, have significant effects on individual and social aspects and can affect daily activities [2]. Tooth loss is a serious life event. Based on WHO criteria, tooth loss is a physical disorder that impairs two important functions, including eating and speaking [3].

In the elderly and young people, tooth loss has significant side effects on different aspects of quality of life, and these effects are internalized by the person [2]. Studies demonstrate that edentulous conditions have negative effects on oral health-related quality of life (OHRQoL), including inability to chew, trouble speaking, and pain and dissatisfaction associated with appearance. Dental implants have beneficial effects in individuals who have lost their teeth [4].

The dentist’s goal is to provide oral health rehabilitation to patients using a pre-determined model. Major and minor edentulism can prevent patients from performing many tasks. Bone loss affects the beauty of edentulous patients. Given that beauty and comfort in speaking are not fully fulfilled with a removable prosthesis, implants can provide an aesthetically pleasant appearance for the patient. The use of implants has a better prognosis than other alternatives. The use of dental implants is an excellent method to restore teeth and oral tissue. Currently, in many parts of the world, this method is used in different cases in tooth-jaw restoration. In this method, a titanium made artificial root is inserted in the patient's jawbone as the tooth substitute. These patients should be studied from various aspects and have the necessary and sufficient indications for this treatment [5].

Although the exact number of dental implant treatments in our country is not known, evidence suggests that the demand for implant therapy is increasing similar to other countries in the world. A majority of dental specialists are familiar with this technique and introduce it as a proper treatment. The introduced process has been accepted worldwide but there are some conditions that may prevent the installation of dental implants like financial or anatomical problems and this may affect the quality of life of the affected person. To assess how a dental implant can affect the daily performances of a patient, a scientific investigation on the influence of the implant in patients’ lives and their satisfaction of the result should be performed. However, some evidence suggests a possible role of implants in quality of life [6].

Given the importance of this subject, the present study aimed to compare the quality of life of patients requesting dental implants before and after implant in Mashhad in 2015.

## 2 MATERIALS AND METHODS

In this cross-sectional study, the oral impact on daily activity (OIDP) index questionnaire was used. This questionnaire was previously validated as a data collection tool for the Iranian population [7]. In the current study, simple random sampling was used. The study was conducted on 73 patients referred to the Mashhad Faculty of Dentistry and dental clinics in 2015. In this study, patients were examined based on the patient's own consent. OIDP includes questions about main daily activities, such as eating, talking, tooth brushing, possible dentures, performing light physical activity, going out, sleeping, relaxing, smiling, emotional stability, enjoying communication with other people, and performing their job. In summary, this study was conducted as follows: the patients were asked if they encountered any potential problems during the last 6 months and expressed their answer as yes or no. Then, the patients were asked about regular or periodic dental problems. The patient’s response to questions regarding the frequency and intensity of problems were scored based on the questionnaire: questions 3 and 4 evaluated the frequency and severity with a score of 1 indicating the lowest frequency and severity. Responses with increased frequency and severity were scored as 2, 3, 4, and 5. The patient's response to question 3 was scored as 1 (less than once a week), 2 (less than once per month), 3 (almost 1-2 time(s) a week), 4 (3 to 4 times a week), and 5 (almost every day). The data were entered in the column for question 3. Regarding question 4, scores were defined as follows: 1 (for 5 days or less), 2 (6 days to one month) 3, (1 to 2 months) 4, (for 2-3 months), and 5 (over 6 months). Then, if the patient's problem affected an activity, its score was recorded as follows: zero (no effect), 1 (very low effects), 2 (relatively low effects), 3 (moderate effects), 4 (relatively severe), and 5 (very severe impact), or 9 (do not know). Data were recorded in the severity section of question 5. For each of these effects, the amount (quantitative data) and the frequency and severity (qualitative data) were recorded. (Quantitative data were assessed qualitatively). In addition, regarding the general health, oral health and its relationship to public health, questions regarding satisfaction with pain in the mouth were asked. For each daily activity Performance score was calculated in this way: (Performance Score=Severity Score × frequency Score) and The OIDP score was determined using this formula:

OIDPpercentage=Sum(multiply)maxpossiblityscore∗100

 

max possiblity score=11×5×5=275

The questionnaire was completed before treatment and one month after implant prosthesis. Finally, the OIDP scores of the patient before and after implants were analyzed using appropriate statistical tests.

The protocol was approved by ethical committee of Mashhad University of Medical Sciences and recorded with this number: IR.mums.sd.REC.1394.85 Statistical analysis was performed using statistical software IBM SPSS version 11.5, Chicago, USA. Kolmogorove- Smirnov test was used to test the normal distribution of data., Kruskal Wallis, Mann-Whitney, Wilcoxon and McNemar tests were used to analyze the data. Levels less than 0.05 were considered significant.

## 3 RESULTS

The study included 73 patients (42 men and 31 women, mean age: 48.72 ± 10.22) who were referred to the dental clinics at the Faculty of Dentistry in Mashhad in 2015.All patients had waited 4-6 months for rehabilitation after implant surgery. As presented in Table **1**, the range of changes was greater in men than in women. In addition, mean changes were greater in men than in women, but this difference was not statistically significant. (*P* = 0.420)

The mean and SD of percentages of OIDP changes before and after surgery in terms of gender indicate that 42 participants were men, and 31 were women (73 participants in total). The results of the Mann-Whitney test revealed no significant differences in mean changes between men and women (*P* = 0.420).

The results of assessing the employment status of 73 cases revealed that 46% were in uncertain job situations, 32% were employees, 34% were members of households, and 34% had other jobs. Table **2** demonstrates that the greatest change was observed in other jobs, housewives and employees. No statistically significant differences were noted among changes in three occupational groups (*P* = 0.489).

The mean and SD of percentages of OIDP changes before and after surgery in terms of jobs are presented in Table **1**. OIDP differences were compared between occupational groups. Kruskal-Wallis test results revealed no statistically significant differences among changes in the three occupational groups (*P*=0.489).

As noted in Table **3**, the range of changes was increased in individuals residing in Mashhad compared with residents of other cities. Moreover, the results indicated that mean changes in residents of cities other than Mashhad were greater than the mean changes in people living in Mashhad (*P* = 0.035).

The mean and SD of percentages of OIDP changes before and after surgery in terms of residence are presented in Table **3**. The Mann-Whitney test results revealed a significant difference between mean changes in non-residents in Mashhad compared with residents of Mashhad (*P*=0.035).

Another variable measured in this study was OIDP changes in terms of educational level. The results of assessing educational level revealed that 15.06% had unknown education, 19.17% were illiterate, 9.58% had secondary school education, 21.9% had high school diploma and associate degree, 24.65% had a bachelor degree, and 9.57% had a master or doctorate degree (73 participants in total). The results of Table **4** demonstrate that the range of changes in high school graduates or lower (non-academic) was considerably greater than that of academically educated participants, but mean changes in these two groups were not statistically significant (*P*=0.754).

The mean ± SD of percentages of OIDP changes before and after surgery in terms of education are presented in Table **4**. The Mann-Whitney test results did not indicate a significant difference between mean changes in both groups (high school or higher or high school or lower) (*P*=0.754).

In this study, OIDP changes were also evaluated between different treatment plans, including overdentures, more than two implants, and one implant. The results of the comparison of OIDP changes between different plans demonstrate that the range of changes and mean changes were as follows: the treatment plan with more than two implants had the greatest range of changes, and the single implant plan had the fewest changes. Mean changes were significant among the three plans, including overdentures, more than two implants, and one implant (*P* = 0.034). On the other hand, when three treatment plans were compared pairwise, the results indicated that the changes in the treatment of more than two implants were significantly increased compared with a single implant, but other pairwise comparisons were not significantly different (Table **5**).

The mean ± SD percentages of OIDP changes before and after surgery based on treatment plan are presented in Table **5**. The Kruskal-Wallis test results revealed a significant difference between mean changes among three plans, including overdentures, more than two implants, and one implant (*P* = 0.034).

Table **6** compares each of the studied conditions before and after surgery. Sleep and resting problems were not significantly reduced, whereas other functional problems significantly decreased after surgery. In addition, the results of this study indicated that the mean OIDP was significantly reduced after surgery compared with before surgery. (*P*˂0.001) (Table **7**).

The frequency distribution of each of the conditions before and after surgery and their comparisons are presented in Table **6**. McNemar test results indicate that most conditions decreased significantly after surgery, except problems with sleeping and resting.

A comparison of OIDP before and after surgery in general is presented in Table **7**. The Wilcoxon test results revealed that the mean OIDP significantly decreased after surgery. (*P*˂0.001)

## 4 DISCUSSION

Concerns about the impact of dental treatment on the patients’ quality of life are increasing. Using social dental indicators to assess quality of life and patient satisfaction have been recently suggested as an important tool for treatment plans because these indicators provide behavioral data in addition to mechanical principles [8]. The assessment tool used in most studies to assess the effect of implantation on a patient’s quality of life is the Oral Health Impact Profile (OHIP) [4, 9-12]. However, OHIP is not validated in Farsi and could therefore not be used for the study. However, a validated Persian version of the OIDP is available [7]. The OIDP is an effective relevant tool that is theoretically concise and accurate. The OIDP estimates that determining the effects caused by oral conditions are focused on the person's ability to perform daily activities and individual behavior [13].

The OIDP is one of the most important OHRQL criteria that considers the impact of oral health on a patient’s ability to perform everyday activities. This index reviews 8 items, including mental, physical and social life [7].

These items include the effect of the mouth on eating; speaking clearly; cleaning teeth or dentures; light physical activities, such as working at home; smiling and showing teeth without discomfort and embarrassment; sleeping and relaxing; enjoying communication with others; performing job-related activities; and emotional conditions, such as getting upset quicker than usual. This tool effectively evaluates the effect of oral health on the quality of life among populations because it is easy to understand and is a short index that does not take much time to complete [1].

The present study evaluated the OIDP index in 73 patients who received implants. Among all patients, 57.8% were male, and 42.2% were female. The mean age of the subjects was 48.72 ± 10.22 years, ranging from 17 to 72 years. Of these subjects, 32% were employees, 34% were housewives, and 34% had other jobs. In this study, 80.3% of participants lived in Mashhad, and 19.3% lived elsewhere. In addition, 91.5% were married, and 8.5% were single. Moreover, 58.1% had high school or lower education, and 49.1% had an associate degree or higher.

Previous studies demonstrated that aging and lower education significantly enhance the effect of oral health on daily living activities, whereas no significant differences were noted between different sexes for the level of the effect of oral health on everyday activities. In addition, education level decreased the therapeutic effects on physical activity. In addition, some studies reported no significant association between socioeconomic status and the effect of oral health on quality of life [1, 14].

In this study, which is with numerous other studies performed in this field, when the number of teeth is reduced, chewing food becomes more difficult. Thus, patients may avoid food and limit their dietary choices [3]. In the present study, the most common problem before surgery was eating (78%). After surgery, the most common problem was smiling, laughing and showing teeth without discomfort and embarrassment (53%). The quality of life associated with eating; speaking clearly; cleaning teeth or dentures; light physical activity; being outside of the home for work or meeting others; smiling, laughing, or showing teeth without discomfort and shame; emotional conditions, such as becoming upset quicker than usual; enjoying communication with other people, such as friends, relatives, and neighbors; and performing their job significantly increased after surgery. However, the quality of life associated with sleeping or resting did not improve.

The results of previous studies demonstrated that the quality of life associated with eating, speaking clearly and communicating with others improved after implantation. However, cleaning teeth, emotional connections and performing daily activities and physical activities were not altered [15]. The results of this study are consistent with the results presented. Following these results, other studies also reported favorable impacts on quality of life for implantations other than dentures in toothless people of different ages [16]. Fillion *et al.* [17] also reported favorable effects of implant on OHRQoL of patients in three areas (functional, psychological, discomfort and pain), and the results suggested that all patients were satisfied with chewing ability and their beauty after treatment. It should be noted that personality of people affects the level of satisfaction with the implant [18]. Other studies have also reported favorable effects of implantation on OHRQoL that underscore the significance of this issue [12, 19]. The results of the present study reveal no significant association between QOL after implants with place of residence, educational level, and sex.

Other studies along with the present study have also reported no significant association between QOL before and after treatment with sex, age, periodontal treatment, and smoking; however, the questionnaire used was different [1].

## CONCLUSION

The quality of life associated with eating; speaking clearly; cleaning teeth or dentures; performing light physical activity; being outside of the home for work or meeting others; smiling, laughing, or showing teeth without discomfort and shame; emotional conditions, such as becoming upset quicker than usual; enjoying communication with other people, such as friends, relatives, and neighbors; and performing their job significantly increased after surgery. However, the quality of life associated with sleeping or resting did not improve. No significant association was noted between QOL after implant with place of residence, educational level, and sex.

## Figures and Tables

**Table 1:** Comparison of OIDP changes in terms of gender.

**Sex**	**Number**	**Mean ± SD**	**Median**	**Minimum**	**Maximum**	**Mann-Whitney Test Result**
**Female**	31	6.44 ± 8.67	4.37	-13.45	31.27	Z = 0.806 *P* = 0.420
**Man**	40	10.26 ± 14.25	5.45	-14.55	57.82	

**Table 2:** Comparison of OIDP changes between different jobs.

**Jobs**	**Number**	**OIDP Changes**	**OIDP percent before surgery**	**OIDP percent after surgery**	**Kruskal-Wallis Test Results**
Mean ± SD	Mean ± SD	Mean ± SD
**Employee**	17	7.81 ± 14.70	10.18 ± 14.21	2.37 ± 4.09	X2 = 1.43 *P*= 0.489
**Housewife**	18	6.57 ± 9.34	9.17 ± 7.85	2.6 ± 4.73	
**Other**	18	12.64 ± 15.33	14.10 ± 15.28	1.45 ± 3.88	

**Table 3:** Comparison of OIDP changes in terms of location of residence place.

**Location of Residence**	**Number**	**Mean ± SD**	**Median**	**Minimum**	**Maximum**	**Mann-Whitney Test Result**
**Mashhad**	53	7.30 ± 11.78	4.00	-13.45	57.82	Z = 2.11 *P* = 0.035
**Other**	13	13.96 ± 1495	16:00	-14.55	47.27	

**Table 4:** Comparison of OIDP changes in terms of education.

**Educational Level**	**Number**	**Mean ± SD**	**Median**	**Minimum**	**Maximum**	**Mann-Whitney Test Result**
**High school or lower**	36	9 ± 12.78	5.45	-13.45	57.82	Z = 0.313 *P* = 0.754
**Associate degree or higher**	26	8.70 ± 13.16	5.45	-14.55	47.27	

**Table 5:** Comparison of OIDP changes between different treatment plans.

Type of Treatment Plan	**Number**	**Mean ± SD**	**Median**	**Minimum**	**Maximum**	**Kruskal-Wallis Test Result**
**Overdentures**	29	10.33 ± 12.17	6.91	-0.73	58.18	X2 = 6.78 *P* = 0.034
**More than two implants**	29	11.81 ± 16.64	5.82	-14.55	57.82	
**One implant**	15	2.74 ± 3.96	1.82	-3.64	11:27	

**Table 6:** Comparison of each of the studied conditions before and after surgery.

**No.**	**Function**	**Before Surgery**		**After Surgery**		**Reduction Compared with Pre-Operation**	**McNemar Test Result**
Number	%	Number	%
**1**	**Eating**	57	78	18	26	68.4	˂0.001
**2**	**Speaking clearly**	26	37	7	10	73.1	˂0.001
**3**	**Cleaning teeth or dentures**	20	28	5	7	75.0	0.001
**4**	**Slight physical activity, such as working at home**	14	20	1	1	92.9	0.001
**5**	**Going shopping or meeting others**	10	14	0	0	100.0	0.002
**6**	**Sleeping**	8	11	4	6	50.0	0.453
**7**	**Resting**	4	6	2	3	50.0	.688
**8**	**Smiling, laughing, or showing teeth without discomfort and shame**	38	53	2	3	94.7	˂0.001
**9**	**Emotional conditions, such as getting upset earlier than usual**	17	24	1	1	94.1	˂0.001
**10**	**Enjoying communication with others, such as friends, relatives, neighbors**	20	29	1	1	95.0	˂0.001
**11**	**Job-related activities**	8	11	0	0	100.0	˂0.00 8

**Table 7:** Mean OIDP before and after surgery.

	**Number**	**Minimum**	**Maximum**	**Mean**	**Standard Deviation**	**Wilcoxon Test Result**
**OIDP before surgery**	73	0.00	60.00	11.1333	12.96469	Z = 5.96 *P*˂0.001
**OIDP after surgery**	73	0.00	16.73	1.7733	3.73942	
